# A retrospective cohort study on fertility in the Norwegian Coldblooded trotter after artificial insemination with cooled, shipped versus fresh extended semen

**DOI:** 10.1186/s13028-015-0161-8

**Published:** 2015-11-14

**Authors:** Caroline Sorknes Haadem, Ane Nødtvedt, Wenche Farstad, Ragnar Thomassen

**Affiliations:** Department of Production Animal Clinical Sciences, Faculty of Veterinary Medicine and Biosciences, Norwegian University of Life Sciences, P.O. Box 8146, 0033 Oslo, Norway

**Keywords:** Norwegian Coldblooded trotter, Artificial insemination, First cycle pregnancy rate, Cooled shipped semen, Fresh extended semen

## Abstract

**Background:**

Pregnancy rates with cooled equine semen can be unsatisfactory and show great variation. Information about first cycle pregnancy rates and pregnancy rates per cycle are often lacking from publicly available records. This retrospective cohort study was performed to evaluate the fertility of the Norwegian Coldblooded trotter. The aim of the study was to compare the breeding results after insemination with fresh, extended with those of cooled, shipped semen among Norwegian Coldblooded trotter mares. First cycle pregnancy rate was the main parameter used to measure fertility. Stud-books were collected from four studs from the years 2006–2010. Statistical analyses were done in Stata using Chi square test and multivariable analyses where different models were compared based on Akaike’s information criterion.

**Results:**

First cycle pregnancy rate, seasonal pregnancy rate and foaling rate all showed significant differences (*P* < 0.0001) when comparing mares inseminated at stud with mares inseminated with cooled, shipped semen, favoring artificial insemination (AI) at stud. First cycle pregnancy rate was 55.1 % for mares inseminated at stud with fresh extended semen and 42.2 % for mares inseminated with cooled shipped semen. The overall pregnancy rate per cycle was 84.4 % for AI at stud and 66.9 % for cooled, shipped semen. The parameters stud, mare age, number of inseminations within an estrus cycle and individual stallion were also investigated for influence on fertility.

**Conclusions:**

Few retrospective studies include the parameter of first cycle pregnancy rates. Our study does not differ dramatically when comparing seasonal pregnancy rates and foaling rates with similar studies. Fertility parameters for the Norwegian Coldblooded trotter do not differ significantly from most other studies of Coldblooded mares and other mare breeds around the world. But the difference in fertility parameters between AI at stud to AI with cooled semen between our study and others, indicates that higher pregnancy rates in Norwegian Coldblooded trotter may be possible.

## Background

Artificial insemination (AI) has been widely used after its introduction in the horse breeding industry. Higher pregnancy rates have been achieved using AI with fresh semen at stud than with natural/hand mating [1, 2]. This is known to be especially true for problem mares [3]. Seasonal pregnancy rates as well as foaling rates vary considerably between different studies. Pregnancy rates with cooled equine semen can be unsatisfactory and show great variation. Jasko et al. [4] reported first cycle pregnancy rates of 76 and 65 % for fresh and cooled semen, respectively. In another retrospective study per-cycle foaling rates after use of cooled semen were approximately 40 % [5]. Management practices may vary among studs; differences in handling, cooling and shipping procedures together with variable semen quality of different stallions make fertility data difficult to compare between regions and individuals. Individual animals considered valuable may get preferential treatment. Publicly available records regarding equine fertility provide information on annual foaling rates for reported matings, however, first cycle pregnancy rates and pregnancy rates per cycle are lacking. Several studies have compared fertility results between fresh, cooled and frozen semen; however, rates reported are mainly based on studies conducted in single or a few stud practices, which may limit the applicability and comparative value of the findings. A survey of the reproductive efficiency of trotters in Finland was published by Katila et al. (2010), where an overall foaling rate of 64 % was reported for the Finn horse. The authors found AI at stud to be the most successful method of mating practice, the difference in foaling rates between AI at stud and AI after transport being three percentage points in favor of AI at stud [6]. Declining foaling rates in the Standardbred and in the Finn horse were reported over the years 1991–2005 [7]. Reported annual foaling rates from the Norwegian Trotting Society (NTS) have been relatively stable since the year 2000; around 67 %. The fertility status of the Coldblooded trotter in Norway is generally assumed to be good, although systematic investigations are unavailable.

There are two breeds used for trotting in Norway; the Standardbred and the Norwegian Coldblooded trotter. The Norwegian Coldblooded trotter and North-Swedish trotter were formally accepted to be one population in the year 2000 by the trotting associations from both countries [8]. Semen is often cooled and shipped between these countries, and a stallion may serve a maximum of 80 mares per year in his home country and 30 mares in the neighboring country. With improvements in transport of cooled semen, mare owners can choose the most popular stallions which may otherwise be unavailable geographically. This has led to a high demand for certain stallions. In Norway peak breeding season is from late May through mid-July. Foaling rate per year is available from NTS. All matings of trotters must be reported from stud managers once a month to NTS. If a mating is not reported, the resulting offspring will not be registered and is not allowed to race. There has been a decline in the number of breeding stallions from 102 registered breeding stallions in the year 2000–64 stallions in the year 2013. The number of registered matings has subsequently decreased from 1821 in the year 2000–915 in 2013 (personal communication, NTS).

There are currently no studies available on the pregnancy rates among Coldblooded trotters in Norway where different insemination practices are compared. There is no information available on seasonal pregnancy rates and therefore no information on the pregnancy loss rate. Nor is there information on first cycle pregnancy rates. Hence, reliable estimates on fertility status in the Norwegian Coldblooded trotter are missing.

The aim of this study was to investigate the difference in breeding results after insemination with fresh or with cooled, shipped semen among Norwegian Coldblooded trotter mares. The primary outcome was the effect of semen cooling and transport on first cycle and seasonal pregnancy rates. Furthermore, pregnancy loss rates were compared between the two groups.

## Methods

### Data source

Records of reported matings of Coldblooded trotter mares covered by Coldblooded trotter stallions during the years 2006–2010 were received from NTS. Both mare and stallion were located in Norway. The original dataset included 4833 breeding episodes, and contained information about stud, stallion and mare identity, whether natural cover or AI was performed and foaling outcome. Studs having >50 covers by AI for 2 or more years were contacted in order to obtain additional data on individual AI covers. Of the initially ten contacted studs, four studs could retrieve their stud books and were willing to share the information. The information obtained from stud books included method of AI (at stud or cooled, shipped), the number of estrus cycles the mares were inseminated and number of inseminations within each cycle. Final outcome of insemination after one season was gathered from the original dataset from NTS, from the NTS website or from mare journals. The data included for analysis collected from studbooks consisted of 1832 breeding episodes. Twenty mares were excluded from further analysis because of missing data.

### Study design and outcome

A retrospective cohort study was performed among mares inseminated from stallions at the four participating studs, to investigate fertility after AI at stud versus AI after shipping cooled semen. Each mare bred by one stallion during one season, by AI either at stud or with cooled shipped semen, was the unit of observation, and the study outcome was the breeding-result. There were seven possible breeding-results in the NTS dataset; live foal, dead foal, foal died after birth, abortion, aborted twins, resorption or non-pregnant. To obtain the foaling rate and the foal loss ratio, breeding-results were divided into three groups; foal (live, dead and died postpartum), lost foal (abortion, aborted twins and resorption), and non-pregnant. Seasonal pregnancy rate and first cycle pregnancy rate were calculated by dividing breeding result into two groups; pregnant (all foaled, aborted and resorbed) or non-pregnant.

### Explanatory variables

First cycle pregnancy rate is the main measurement of fertility in this study. It comprises all mares pregnant after AI in the first cycle they were bred that season. Per cycle pregnancy rate is the total number of mares pregnant over the total number of breeding cycles. Seasonal pregnancy rate is the ratio of pregnant mares at the end of the season, regardless of number of cycles they were inseminated. All mares that did deliver a foal (live or dead) at term, the next season, divided by total numbers of mares bred is the foaling rate. Method of insemination was either *AI with fresh, extended semen at stud* or *AI with cooled, shipped semen*. The age of stallions and mares at the year of insemination was calculated from their year of birth, obtained from their id or the horse register available at the website of NTS. Mares were divided into three age groups– young; 2–10 years of age, mature; 11–15 years of age and older; 16–24 years of age. The studs were each given a letter A–E. Some stallions from other studs than the four from where studbooks were collected were also included, as the four studs sometimes received cooled semen shipped from elsewhere. These other studs were grouped together for the statistical analyses.

### Statistical analysis

#### Descriptive analysis

The data files were originally obtained in Excel. These were checked for inconsistencies and missing data before they were imported into Stata (Stata SE/12, Stata Corp., College Station, TX, USA) for descriptive statistical analyses. Further data management and statistical analyses were performed in Stata. First cycle pregnancy rates, seasonal pregnancy rates, pregnancy loss rates and foaling rates were compared between fresh and cooled semen using Chi square test. Cut-off for statistical significance was set to *P* ≤ 0.05.

#### Multivariable analysis

The relationship between first cycle pregnancy rate and method of semen handling was investigated further using a random effects logistic regression model. The confounding variables “age of mare” (three levels), “stud”, “year” and “stallion” were tested in the model as fixed effects. Mare was included as a random variable to control for the lack of independence between observations from the same mare across different breeding seasons. Furthermore, a model where the effect of mare was ignored and stallion was included as a random variable was built for comparison. The cut-off for keeping fixed effect variables in the model was set to *P* < 0.05 and different models were compared based on Akaike’s information criterion (AIC).

## Results

### Descriptive findings

A final number of 1812 breeding episodes were available for analysis. AI on stud with fresh extended semen was reportedly used in 1257 breeding episodes and AI with cooled, shipped semen was reported in 555 breeding episodes. A total of 1092 mares made up the breeding episodes (observations per mare 1–6, mean 1.7). Mares ranged from 2–24 years of age during the year of insemination. Age distribution of mares did not differ between those receiving fresh semen and those inseminated with cooled, shipped semen. This was controlled by Chi square test in Stata, using mare age groups. Semen from 21 different stallions was used. The stallions ranged between 4 and 15 years of age and were mainly located at 4 different studs. The stallions served a mean of 86.1 mares per season. A slight increase in the proportions between use of cooled, shipped semen AI and at stud AI during the last 2 years was observed.

#### First cycle pregnancy rate, seasonal pregnancy rate and foaling rate

First cycle pregnancy rate, seasonal pregnancy rate and foaling rate all showed significant differences (*P* < 0.0001) when comparing mares inseminated at stud with mares inseminated with cooled, shipped semen, favoring AI at stud (Table 1). Differences between the two categories of AI were 12.9 percentage points for first cycle pregnancy rate, 17.5 percentage points for seasonal pregnancy rate and 16.4 percentage points for foaling rate, all in favor of AI at stud with fresh extended semen (Table 1). The overall foaling rate for all the years regardless of AI with fresh or cooled semen was 74.3 %, whereas the live foal rate was 73.0 %. Seasonal pregnancy rate was 79.0 %, giving a pregnancy loss rate of 4.7 %.

**Table 1:** Differences between variables on mare fertility after AI with fresh extended versus cooled transported semen

Semen used	No. of mares		1st cycle pregnancy rate		Seasonal pregnancy rate		Pregnancy loss rate		Foaling rate	
	Fresh	Cooled	Fresh	Cooled	Fresh	Cooled	Fresh	Cooled	Fresh	Cooled
Mare age										
Younger (2–10)	625	271	57.4^a^	46.7^b^	87.0^c^	70.9^d^	4.6	4.1	82.4^e^	66.8^f^
Mature (11–15)	416	193	55.5^a^	38.3^b^	84.1^c^	64.8^d^	5.5	4.2	78.6^e^	60.6^f^
Older (16–24)	216	91	47.7^a^	37.4^a^	77.3^c^	59.3^d^	5.6	3.3	71.8^e^	56.0^f^
Stud										
A	763	389	51.4^a^	44.0^b^	83.6^c^	69.4^d^	5.9	3.3	77.7^e^	66.1^f^
B	158	54	58.2^a^	20.4^b^	87.3^c^	51.9^d^	3.2	3.7	84.2^e^	48.2^f^
C	131	46	58.0^a^	50.0^a^	87.0^c^	76.1^c^	3.0	6.5	84.0^e^	69.6^e^
D	205	58	64.9^a^	44.8^b^	83.4^c^	60.3^d^	4.9	6.9	78.5^e^	53.5^f^
E	0	8	–	37.5	–	37.5	–	0.0	–	37.5
Year										
2006	137	75	54.0^a^	44.0^a^	81.8^c^	65.3^d^	4.4	5.3	77.4^e^	60.0^f^
2007	243	81	47.3^a^	46.9^a^	84.0^c^	69.1^d^	4.1	2.5	79.8^e^	66.7^f^
2008	368	103	57.9^a^	28.2^b^	84.0^c^	57.3^d^	4.6	1.6	79.4^e^	55.3^f^
2009	297	158	58.9^a^	51.9^a^	86.5^c^	76.6^d^	4.4	7.0	82.2^e^	69.6^f^
2010	212	138	54.7^a^	37.7^b^	84.4^c^	62.3^d^	8.5	2.2	75.9^e^	60.1^f^
Overall	1257	555	55.1^a^	42.2^b^	84.4^c^	66.9^d^	5.1	4.0	79.3^e^	62.9^f^

All numbers are given in %. Values between fresh and cooled semen on fertility parameters within a row with different superscripts vary significantly

#### Breeding cycles and inseminations

**Fig. 1:**
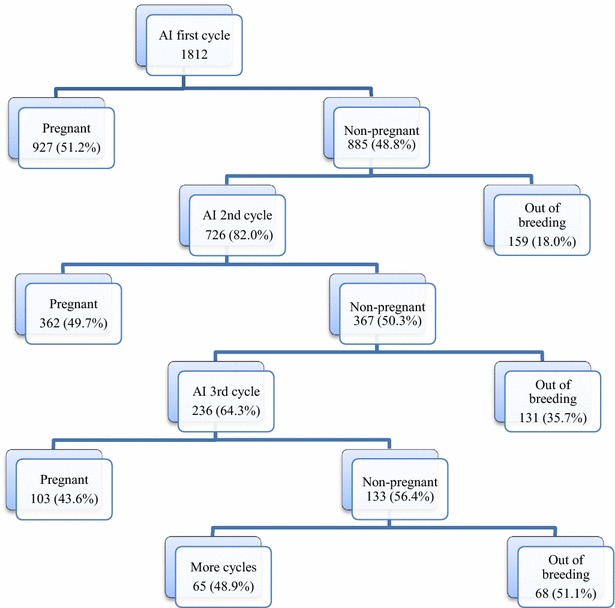
Flow diagram showing the breeding cycles of mares. Numbers represent mares, percentages are given in brackets

Information on the number of cycles per breeding season was also obtained. The dataset from studs consisted of altogether 2843 inseminated estrus cycles, of which 1945 (68.4 %) cycles were by AI with fresh extended semen and 898 (31.6 %) cycles were by AI with cooled, shipped semen, respectively. The overall pregnancy rate per cycle was 84.4 % for AI at stud and 66.9 % for cooled, shipped semen (*P* < 0.0001). Overall per cycle foaling rate was 79.3 % for fresh semen and 62.9 % for shipped semen (*P* < 0.0001). One cycle pregnancy rate was 54.7 and 40.8 % for AI at stud and AI with cooled, shipped semen, respectively (*P* < 0.0001). Numbers in brackets are calculated using Chi square test. No mare was bred for more than five cycles. See Fig. 1 for a flow diagram over number of breeding cycles. Of the 927 mares that conceived in their first breeding cycle, 850 (91.7 %) gave a live foal next season, giving a first cycle live foaling rate of 47 %. The pregnant mares that did not produce a live foal either resorbed 24 (2.6 %), or aborted 37 (4.0 %), or had a stillbirth or a foal that died immediately postpartum 16 (1.7 %). Live foaling rate for second cycle was 46.3 %, as 24 (6.7 %) of the pregnant mares did not produce a live foal.

The highest number of inseminations in one inseminated estrus cycle was eight, while total number of inseminations did not exceed 14 in any mares. The majority of mares (n = 1024) was inseminated 2–3 times during one estrus cycle. Mares that were inseminated more than once were favored for all parameters on fertility (first cycle pregnancy rate *P* = 0.039, seasonal pregnancy rate *P* = 0.003 and foaling rate *P* = 0.002).

#### Age

Increasing mare age had a negative effect on first cycle pregnancy rates (*P* = 0.013). Mares of higher age showed an increased tendency of being non-pregnant at the end of season (*P* = 0.001) and a decreased tendency of a foal born (*P* = 0.003). Live foal rate was 76.8 % in mares aged between 2 and 10 years, 70.6 % in mares aged 11–15 years, and 65.8 % in mares aged 16–24 years. See Table 1 for details on fertility parameters between age groups. Fertility parameters did not show significant differences between mare age groups when only considering mares receiving cooled, shipped semen. Stallion age had no effect on fertility parameters in this study.

#### Stud

First cycle pregnancy rate showed variation between studs ranging from 37.5 to 60.5 % (*P* = 0.001), regardless of method of AI. There were also variations between studs when comparing first cycle pregnancy rate with insemination practice (AI at stud vs. AI with cooled, shipped semen). First cycle pregnancy rates for all studs are given in Table 1.

#### Stallion

Foaling rate varied between stallions. A few stallions served only between one and seven mares during the period of this study, these were mostly stallions kept at other studs than where the studbooks were obtained. When excluding stallions with less than 10 covers, first cycle pregnancy rate for individual stallions varied from 17.7 to 73.0 %. The foaling rate between individual stallions varied from 35.3 to 87.9 %.

### Multivariable analysis

The final model included the variables “age of mare”, “stud” and “stallion” in addition to “method of semen handling” and the random mare-effect. Due to missing data for some variables, the model was based on 1809 observations from 1092 mares (observations per mare 1–6, mean 1.7). The odds ratio (OR) for first cycle pregnancy based on this model was 1.68 (*P* < 0.001) for fresh compared to cooled, shipped semen. The odds of first cycle pregnancy decreased by increasing age and mature and older mares had odds ratios of 0.85 (0.67–1.07) and 0.64 (0.47–0.86), respectively, compared to young mares. The intra-class correlation coefficient (rho) for mare was 0.08 and highly significant (*P* = 0.02), which means that 8 % of the variation in first cycle pregnancy rates could be attributed to the effect of mare in this model. In the model where stallion was included as a random effect, stud no longer had a significant effect on first cycle pregnancy rate based on comparison of likelihood ratio and AIC, but the OR for method of semen handling remained virtually unchanged.

## Discussion

This retrospective cohort study quantified the effect of AI with cooled, shipped versus fresh extended semen on first cycle pregnancy rates in Norwegian Coldblooded trotter mares.

In this study first cycle pregnancy rate was the main focus. This will give the most valuable information on fertility, since “problem mares” that are unlikely to get pregnant during consecutive cycles will not influence statistics as much.

Studies on stallion fertility often use the parameter of first cycle pregnancy rate, minimizing the effect of mares and management that might negatively affect fertility [9]. We found variations between all fertility parameters when comparing AI at stud with AI with cooled, shipped semen. First cycle pregnancy rate had a difference of 12.9 percentage points in favor of AI at stud. In another retrospective study pregnancy rates per cycle were reported to be 77 % for fresh and 43 % for cooled semen, and 56 % for fresh and 45 % for cooled semen, in the years of 2002 and 2003, respectively [10]. Another study reported first cycle pregnancy rates of 59 % in mares inseminated with cooled semen [11]. Rota et al. (2004) reported first cycle pregnancy rates in Maremmano mares of 42.8 % for cooled semen. For our study, the odds of pregnancy during first cycle were 68 % higher among mares inseminated with fresh compared to cooled, shipped semen. This was after controlling for lack of independence between observations from the same mare as well as for the confounding effects of age, stud and stallion. Based on the alternative model with a random stallion-effect it appears that when controlling for stallion in this manner, stud becomes insignificant. This would suggest that the observed variation in first cycle pregnancy results between studs could be explained by differences between stallions.

Seasonal pregnancy rate after AI with cooled semen was 67.8 % in one study [9]. Crowe et al. [12] reported seasonal conception rate for cooled semen of 69.6 % with one pre-ovulatory insemination after the use of deslerolin acetate to induce ovulation. This was a retrospective study with a variety of breeds from four studs, but AI method was standardized and also included treatment after AI with oxytocin and intrauterine (IU) antibiotics. Fertility parameters are prone to be influenced by factors such as stud, management and individuals. The study by Rota et al. [9] showed a difference of 28.6 percentage-points in cooled semen seasonal pregnancy rate when comparing AI at stud centers to AI at home in less controlled environment, in favor of AI centers. In our study the seasonal pregnancy rate was 66.9 % for cooled shipped semen. However, there was no standardization for how the AI was done here, because this study was performed as an observational rather than an experimental study.

Katila et al. [6] found a difference in foaling rates of the Finn horse of three percentage points in favor of AI at stud compared to cooled, shipped semen. In our study the difference in foaling rate was 16.4 percentage points in favor of AI at stud compared to AI with cooled, shipped semen. Overall foaling rates also differed between these two studies (64 % in the Finnhorse and 74.3 % in the Coldblooded trotter). Reported foaling rates from NTS of the Norwegian Coldblooded trotter is around 67 %. Foaling rates from our study are around ten-percentage point better compared to foaling rates reported from the Finn horse in the years 1991–2005, as well as for figures reported from NTS. This could be explained by the quality and experience of the studs from where the material was gathered in our study. Live foal rates were reported to be 70 % in a retrospective part of a study using semen cooled for 12 h before AI [5]. All mares in this study were of warmblood breed and located at the same stud. High foaling—and seasonal pregnancy rates can also be hiding mares inseminated for repeated cycles, like several mares in our study, considering our first cycle pregnancy rate was 51.2 % overall and 42.2 % for cooled semen. Mares inseminated with stallions from “other” studs were very few (n = 8) and these mares only received cooled, shipped semen. Still, the tendency was that mares receiving cooled, shipped semen from one of the “other” studs, had lower first cycle pregnancy rates. Mares receiving cooled, shipped semen from stud B, also showed significantly lower first cycle pregnancy rates (20.4 %), than those from studs A, C and D. There might have been a weakness in the handling of cooled semen at stud B, or the stallion(s) for service at this stud may have been so-called “bad cooler(s)”, hence, semen quality at AI may have been compromised.

The strength of the cohort design is that the results are often representative for the situation in the field, but the trade-off is a lack of standardization compared to controlled experiments. Treatment practices as well as AI practices vary greatly, not only between studs, but also between different breeds and between countries. Few retrospective studies include the parameter of first cycle pregnancy rates. Our study does not differ dramatically when comparing seasonal pregnancy rates and foaling rates with similar studies. However, it would be more useful if the variable of comparison was the first cycle pregnancy rates, for reasons mentioned before.

In observational studies factors that might have a great effect on results are not randomized, as they would be in a clinical trial. To avoid confounding variables from unduly influencing the results, a regression model was built controlling for the effects of stallion, stud, year and age of the mare. In this study we could conclude that age had no influence on distribution between groups or the first cycle pregnancy rate and seasonal pregnancy rate within and between groups. However, there was no information available on reproductive status of the mares, and thus there could be more barren mares in one of the groups (fresh/cooled semen). Other studies have also shown that foaled mares and maiden mares are likely to have better fertility compared to barren or open mares [13, 14]. If “problem” mares are more likely to be sent to and inseminated at stud instead of receiving cooled, shipped semen, this could also be a source of bias as it could give falsely higher pregnancy rates for mares receiving cooled semen. Pregnancy rates after AI with cooled, shipped semen depend on several factors. As well as stallion, mare and stud factors; semen dose, semen handling and cooling, temperature during storing and shipment, extender used, and number of inseminations of the mares will influence conception rates. Our finding that mares with >1 AI per cycle had better conception rates, may indicate that the AI dose is too low or that AI is not done at an optimal time. Because the current study is a retrospective cohort based on secondary data, it was not feasible to gather more information regarding the above-mentioned factors and prospective data-collection would need to be performed to gain more insight regarding their effect on the fertility results.

Because we could only receive stud books from four different studs, much of the information about the breeding of the Norwegian Coldblooded trotter is still missing. A relevant weakness with the limited number of studs could be that only the largest, most efficient studs with long experience and thus, the most popular stallions (possibly receiving the “best” mares), were included in our study. The bigger difference in fertility parameters between AI at stud to AI with cooled semen between our study and others, indicates that higher pregnancy rates in Norwegian Coldblooded trotter may be possible.

Standardizing and optimizing cooling and shipment, as well as ensuring large enough doses will be crucial to get better results after cooled, shipped semen. Further studies on effect of AI doses as well as semen handling and shipment at different studs in Norway would be beneficial.

## Conclusions

Breeding results of the Coldblooded trotter breed in Norway do not differ significantly from most other studies of Coldblooded mares and other mare breeds around the world. There is a marked higher first cycle pregnancy rate after AI at stud compared with AI with cooled, shipped semen.
